# Impact of Serum Indoxyl Sulfate on One-Year Adverse Events in Chronic Kidney Disease Patients with Heart Failure

**DOI:** 10.3390/jcm13154384

**Published:** 2024-07-26

**Authors:** Keiichiro Iwasaki, Toru Miyoshi, Chikara Urabe, Satoru Sakuragi, Yusuke Kawai, Soichiro Fuke, Masayuki Doi, Atsushi Takaishi, Takefumi Oka, Naoto Tokunaga, Hiroshi Ito

**Affiliations:** 1 Department of Cardiovascular Medicine, Okayama University Institute of Academic and Research, Medicine, Dentistry and Pharmaceutical Sciences, Okayama 700-8558, Japan; 2 Department of Cardiovascular Medicine, Iwakuni Clinical Center, Iwakuni 740-8510, Japan; 3 Department of Cardiovascular Medicine, Okayama City Hospital, Okayama 700-8558, Japan; 4 Department of Cardiovascular Medicine, Japanese Red Cross Okayama Hospital, Okayama 700-8558, Japan; 5 Department of Cardiology, Kagawa Prefectural Central Hospital, Takamatsu 760-8557, Japan; 6 Department of Cardiology, Mitoyo General Hospital, Kanonji 769-1695, Japan; 7 Department of Cardiology, Tsuyama Chuo Hospital, Tsuyama 708-0841, Japan; takefumi1009oka@yahoo.co.jp; 8 Department of Cardiology, Ibara City Hospital, Ibara 715-0019, Japan; denhyou@gmail.com; 9 Department of General Internal Medicine 3, Kawasaki Medical School, Okayama 700-8558, Japan

**Keywords:** heart failure, chronic kidney disease, indoxyl sulfate

## Abstract

**Background/Objectives:** Indoxyl sulfate, a uremic toxin, is associated with mortality and cardiovascular events in patients with chronic kidney disease (CKD). This study aimed to evaluate the prognostic implications of serum indoxyl sulfate levels in patients with heart failure and CKD. **Methods and Results:** This was a prospective multicenter observational study. Overall, 300 patients with chronic heart failure with a previous history of hospitalization and an estimated glomerular filtration rate (eGFR) of 45 mL/min/1.73 m^2^ or less (CKD stage G3b to G5) without dialysis were analyzed. The primary outcome assessed in a time-to-event analysis from the measurement of indoxyl sulfate was a composite of all-cause death, hospitalization for heart failure, nonfatal myocardial infarction, and nonfatal stroke. Clinical events were followed-up to one year after indoxyl sulfate measurement. The median patient age was 75 years, and 57% of the patients were men. We divided the cohort into low and high indoxyl sulfate categories according to a median value of 9.63 mg/mL. The primary outcome occurred in 27 of 150 patients (18.0%) in the low indoxyl sulfate group and 27 of 150 patients (18.0%) in the high indoxyl sulfate group (hazard ratio, 1.00; 95% confidence interval, 0.58 to 1.70, *p* = 0.99). In the post hoc exploratory analyses, the results were consistent across age, sex, body mass index, left ventricular ejection fraction, eGFR, and N-terminal pro b-type natriuretic peptide. **Conclusions:** Among heart failure patients with CKD stages G3b to 5G, serum indoxyl sulfate concentrations were not significantly associated with the subsequent occurrence of cardiovascular events.

## 1. Introduction

The heart and kidneys are closely related to the establishment of homeostasis, regulating the circulating blood volume and vascular resistance to maintain adequate blood flow throughout the body. Recently, the bidirectional cross-talk of chronic kidney disease (CKD) and cardiovascular disease has gained widespread recognition, leading to the proposal of cardiorenal syndrome [1,2]. In patients with CKD, there is a systemic accumulation of uremic toxins due to renal excretory dysfunction, affecting various organs, including the kidney and the heart [3]. Indoxyl sulfate, a uremic toxin [4], is known to exert profibrotic, prohypertrophic, and proinflammatory effects in vitro studies [5]. Moreover, indoxyl sulfate is an independent predictor of atherosclerotic cardiovascular events [6] and all-cause mortality [7] in patients with CKD.

Plasma indoxyl sulfate concentrations are higher in patients with heart failure than in healthy individuals without heart failure [8]. Additionally, two previous studies have demonstrated an independent association between indoxyl sulfate levels and the composite of cardiac death or hospitalization for heart failure among patients with chronic heart failure [9,10]. However, as both were retrospective, single-center, observational studies, a prospective multicenter study is needed to validate the predictive value of indoxyl sulfate levels for mortality and cardiovascular events in patients with heart failure. Consequently, this study aimed to prospectively evaluate the association between serum indoxyl sulfate levels and a composite of all-cause death, hospitalization for heart failure, nonfatal myocardial infarction, and nonfatal stroke.

## 2. Methods

### 2.1. Study Population

Eligible participants included ambulatory patients who were aged 20 years or older and younger than 85 years, had chronic heart failure with a previous history of hospitalization, and had an estimated glomerular filtration rate (eGFR) of 45 mL/min/1.73 m^2^ or less (CKD stage G3b to G5) [11]. The exclusion criteria were as follows: history of hospitalization for heart failure within 30 days, treatment with oral spherical carbonaceous adsorbent, undergoing dialysis, diagnosis of nephrotic syndrome, diagnosis of polycystic kidney disease, life expectancy of less than one year, pregnant. Clinical events were followed-up to one year after indoxyl sulfate measurement.

### 2.2. Data Collection and Echocardiography

Baseline characteristics information, including age, sex, previous medical histories, medications, smoking status, alcohol status, blood pressure, heart rate, New York Heart Association classification, complete blood count, biochemistry, left ventricular ejection fraction (LVEF), left ventricular diastolic diameter, left ventricular systolic diameter, interventricular septal thickness, posterior wall thickness, left atrial diameter, e’, E/e’, and tricuspid regurgitation pressure gradient, was collected at a local hospital. Blood samples for indoxyl sulfate measurements were collected at baseline and sent to a core laboratory.

### 2.3. Measurement of Indoxyl Sulfate

Blood samples were collected from patients upon registration in the outpatient department. Laboratory values were determined at the central laboratory in each hospital. Residual serum was separated and stored at −80°. Indoxyl sulfate was measured using the enzyme method (NIPRO Corporation, Osaka, Japan) at the core laboratory of Okayama University, as previously described [12,13]. Briefly, sulfatase converted indoxyl sulfate to indoxyl. The generated indoxyl reacted with the tetrazolium salt to produce formazan, and the level of indoxyl sulfate was calculated according to the change in absorbance at 450 nm caused by the generated formazan.

### 2.4. Definition of Outcome

The primary outcome, assessed using a time-to-event analysis of indoxyl sulfate measurements, was a composite of all-cause death, hospitalization for heart failure, nonfatal myocardial infarction, and nonfatal stroke. Secondary outcomes included all-cause death, hospitalization for heart failure, nonfatal myocardial infarction, nonfatal stroke, and renal composite outcomes. The renal composite outcome was defined as a composite of renal replacement therapy, renal transplantation, and initiation of oral spherical carbonaceous adsorbent. The outcome data were adjudicated using an independent data monitoring board.

### 2.5. Sample Size Calculation

A previous study showed that the annual incidence of hospitalization for heart failure in patients with dilated cardiomyopathy was approximately 30% for the entire cohort [9]. Another study showed that the event rates, including all-cause death or hospitalization in patients with heart failure, were almost 50% in those with an eGFR of less than 30 mL/min/1.73 m^2^ and 30% in those with an eGFR of 30 to 50 mL/min/1.73 m^2^ at one year [14]. Taken together, we hypothesized that the incidence of the primary outcome in the high and low indoxyl sulfate groups would be 35% and 20%, respectively, when the study cohort was divided into two groups based on the median value of indoxyl sulfate. Using a two-sided paired test for differences, a minimum sample size of 284 patients was required to detect statistically significant differences in the primary outcome with a power of 80% and an α-type error of 5%. Assuming a dropout rate of 10%, the target sample size was 312.

### 2.6. Statistical Analysis

All statistical analyses were performed using R version 4.1.2. The study cohort was divided into two groups based on the median value of indoxyl sulfate. Categorical and continuous variables are described as frequencies (%) and medians with interquartile ranges (IQR), respectively. The distribution of each continuous variable was assessed using histograms. Continuous and categorical variables were compared using the Mann–Whitney U and chi-squared tests, respectively. Follow-up time was calculated using the Kaplan–Meier estimate of potential follow-up, and log-rank tests were used to compare clinical outcomes according to the indoxyl sulfate categories. The primary and secondary outcomes were assessed using a univariate Cox regression proportional hazards model. The following subgroups were analyzed for the following primary outcome: age (<median, ≥median), sex, body mass index (<median, ≥median), left ventricular ejection fraction (LVEF) category (LVEF < 40%, LVEF ≥ 40%), eGFR (median, ≥median), and NT-proBNP (<median, ≥median). The proportionality assumptions of the Cox regression models were evaluated using log–log survival curves. Multiple imputations were used to handle missing data. Statistical significance was set at *p* < 0.05.

## 3. Results

Overall, 312 patients at eight sites were enrolled between 1 April 2019, and 30 September 2020. Of these, 18 patients were excluded: 3 patients for duplicate registration, 2 patients who did not meet the eligibility criteria, 11 patients for no indoxyl sulfate measurements, and 2 patients for withdrawal of consent. The data from these 300 patients were included in the final analysis. We divided the cohort into low and high indoxyl sulfate categories according to a median value of 9.63 mg/mL.

Table 1 presents the demographic and baseline characteristics of the two groups. The median patient age was 75 years, and 57% of the patients were men. A total of 64 patients (21%) patients had ischemic heart disease. The median LVEF was 45%, and 121 patients (41%) had an LVEF of <40%. The median eGFR was 34 mL/min/1.73 m^2^. The median indoxyl sulfate values in the low and high groups were 5.85 mg/mL and 14.65 mg/mL. There were no significant differences between the low and high indoxyl sulfate groups, except for a history of diabetes mellitus (*p* = 0.01) and eGFR (*p* < 0.001).

**Figure 1:**
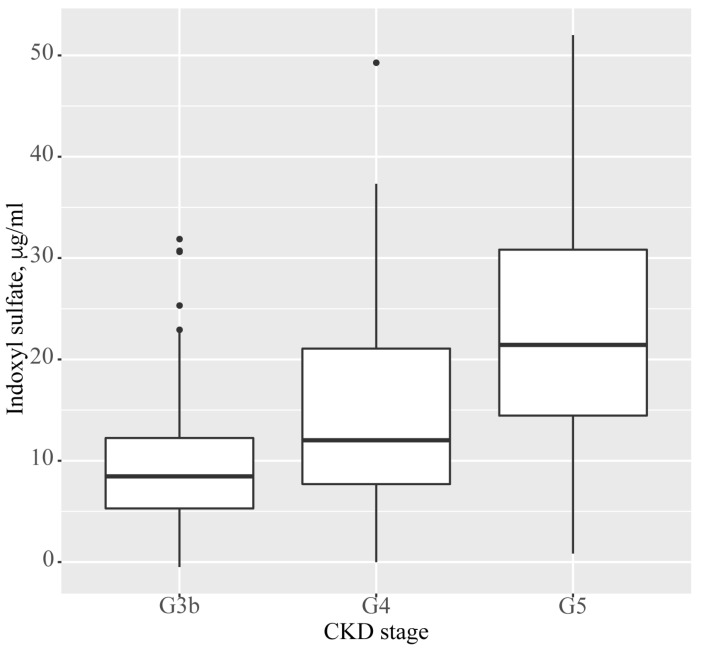
Serum levels of indoxyl sulfate in each CKD stage. The serum levels of indoxyl sulfate are illustrated in CKD stage G3b (eGFR: 30–45 mL/min/1.73 m^2^), stage G4 (eGFR: 15–30 mL/min/1.73 m^2^), and stage G5 (eGFR: <30 mL/min/1.73 m^2^). Abbreviations: CKD, chronic kidney disease; eGFR, estimated glomerular filtration rate.

As described in Figure 1, the median indoxyl sulfate levels in each CKD stage were 8.46 mg/mL (IQR 5.30 to 12.24 mg/mL) in stage G3b, 12.02 mg/mL (IQR 7.40 to 21.06 mg/mL) in stage G4, and 21.43 mg/mL (IQR 14.45 to 30.83 mg/mL) in stage G5.

**Figure 2:**
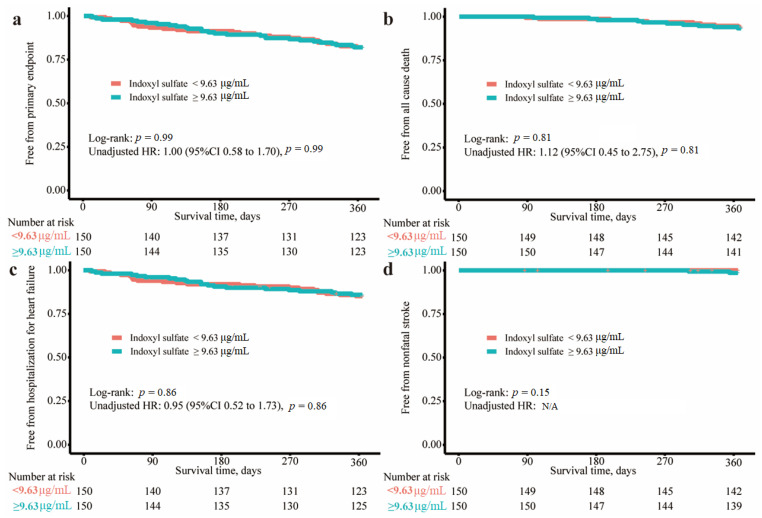
The primary and key secondary outcomes in each indoxyl sulfate group. The event-free survival curves based on the Kaplan–Meier estimator in each indoxyl sulfate group for (**a**) the primary outcome, (**b**) all-cause death, (**c**) hospitalization for heart failure, and (**d**) nonfatal stroke. Abbreviations: HR, hazard ratio; 95%CI, 95% confidece interval.

**Figure 3:**
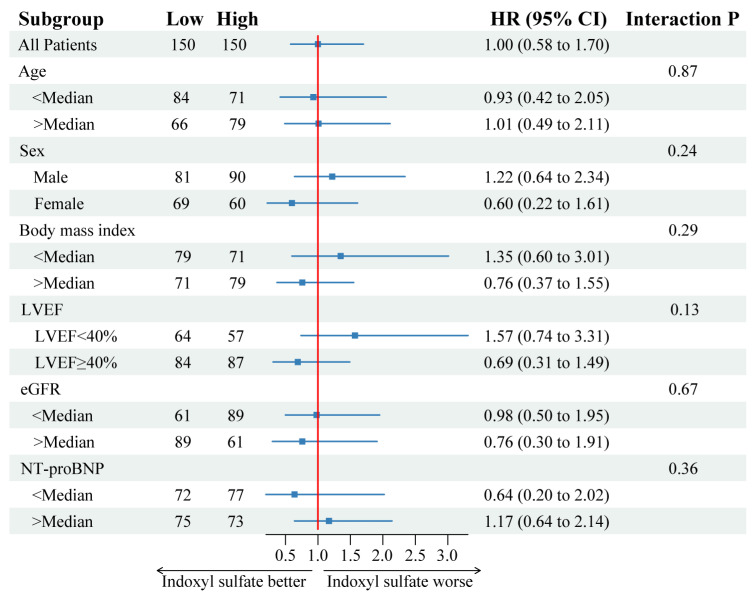
Forest plot of the primary outcome by subgroups. HR, hazard ratio; LVEF, left ventricular ejection fraction; NT-proBNP, N-terminal pro-B-type natriuretic peptide; 95%CI, 95% confidence interval.

The primary outcome occurred in 27 of 150 patients (18.0%) in the low indoxyl sulfate group and 27 of 150 patients (18.0%) in the high indoxyl sulfate group (hazard ratio, 1.00; 95% confidence interval [CI], 0.58 to 1.70, *p* = 0.99), as described in Figure 2 and Table 2. In the post hoc exploratory analyses, the results were consistent across a wide variety of subgroups (Figure 3). The receiver operating characteristic curve analysis for the primary outcome revealed that the area under the curve was 0.53 (95%CI: 0.44 to 0.62). Further analysis of the secondary outcomes was not performed because only one renal composite outcome was documented.

The primary outcome was a composite of all-cause death, nonfatal myocardial infarction, nonfatal stroke, and hospitalization for heart failure. The renal composite outcome was a composite of dialysis, renal transplantation, and initiation of oral spherical carbonaceous adsorbent administration.

## 4. Discussion

This prospective, multicenter, observational study demonstrated that serum indoxyl sulfate concentrations were not significantly associated with the composite of all-cause death, hospitalization for heart failure, nonfatal myocardial infarction, or nonfatal stroke in patients with heart failure and CKD. In the post hoc exploratory analyses, the relationship between serum indoxyl sulfate levels and primary outcomes was consistent across various subgroups in the study cohort. Additionally, there were no significant differences between the low and high indoxyl sulfate groups in terms of echocardiographic functional parameters.

The findings of the current study are not consistent with those of previous retrospective, single-center studies [9,10], which reported a significant independent association between indoxyl sulfate levels and the composite of cardiac death or hospitalization for heart failure and a significant association between indoxyl sulfate levels and echocardiographic cardiac functions. There were several differences between the current study and the two previous studies. Firstly, as the current study targeted patients with heart failure with CKD stages G3b to G5, the eGFR of the study cohort was lower than that of the patients in the previous two studies. The relationship between indoxyl sulfate and clinical outcomes in the early stages of CKD may differ from that observed in our cohort. Secondly, the previous studies enrolled patients who were discharged after treatment for heart failure, whereas our study recruited stable outpatients with a history of heart failure. This difference in patient condition could have affected the association between circulating indoxyl sulfate and clinical outcomes. Finally, this was a prospective, multicenter study. Because previous evidence was limited to single-center, retrospective studies, the results of the present study are considered informative.

Among patients with CKD without heart failure, several studies have indicated a significant association between indoxyl sulfate levels and all-cause death or atherosclerotic cardiovascular diseases [4,6,7]. Although the current study was designed to evaluate a composite outcome, including atherosclerotic cardiovascular disease, the sample size was insufficient to evaluate the effects of indoxyl sulfate on each component of the primary outcome. Our study cohort included only 21% of patients with prior ischemic heart disease at baseline, and two cases of stroke and no myocardial infarction were documented. A patient background of a low risk of atherosclerotic cardiovascular disease may be associated with a low event rate.

Determining whether indoxyl sulfate levels have prognostic biomarker value in heart failure beyond the influence of renal dysfunction is crucial because indoxyl sulfate levels are highly affected by renal function, which is one of the strongest prognostic predictors of clinical outcomes in heart failure [15]. Unfortunately, the findings of the current study did not indicate the value of indoxyl sulfate as a prognostic biomarker. However, it is also important to determine whether interventions to lower indoxyl sulfate levels are beneficial in patients with heart failure or CKD. A small randomized, double-blind, placebo-controlled trial in patients with CKD demonstrated that AST-120, an oral spherical carbonaceous adsorbent, decreased serum indoxyl sulfate levels in a dose-dependent fashion [16]. Moreover, AST-120 administration ameliorated vascular dysfunction in CKD mice models [17]. Further studies are needed to elucidate whether interventions targeted at reducing indoxyl sulfate levels are beneficial in patients with heart failure or CKD.

The subgroup analysis in the current study showed that LVEF did not affect the association between indoxyl sulfate levels and the primary outcome. However, Shimazu et al. reported that serum indoxyl sulfate level was a significant predictor of cardiac events in patients with dilated cardiomyopathy. The reason for this discrepancy remains unclear; however, further investigation is required to ascertain whether indoxyl sulphate has a distinct prognostic impact based on the LVEF.

This study has some limitations. Firstly, the event rate in this study was lower than anticipated. However, as the difference between the expected and actual one-year event rate in the low-indoxyl sulfate group was only 2% (expected 20% vs. actual 18%), the influence of the primary analysis was limited. Secondly, since the follow-up period of the current study was one year, the effects of indoxyl sulfate on clinical outcomes beyond one year are unknown. An enzymatic method was used to evaluate indoxyl sulfate concentrations in the current study. Although high-performance liquid chromatography is the gold standard, enzymatic methods have been reported to correlate well with high-performance liquid chromatography [12,13]. However, we could not exclude the influence of the indoxyl sulfate measurement method. Finally, the use of SGLT2 inhibitors was only 18% in this study; SGLT2 inhibitors have been shown to improve prognosis in patients with heart failure [18]. Therefore, it remains uncertain whether indoxyl sulfate can serve as a biomarker for prognosis in patients with CKD with heart failure under modern guideline-directed medical therapy. Further studies are necessary to elucidate this.

## 5. Conclusions

Among patients with heart failure with CKD stages G3b to 5G, serum indoxyl sulfate concentrations were not significantly associated with the subsequent occurrence of the composite of all-cause death, hospitalization for heart failure, nonfatal myocardial infarction, and nonfatal stroke.

## Figures and Tables

**Table 1:** Baseline demographic and clinical characteristics.

	Overall	Low Indoxyl Sulfate < 9.63 mg/mL	High Indoxyl Sulfate ≥ 9.63 mg/mL	*p*-Value
n	300	150	150	
Age, year	75 [69, 80]	75 [68, 80]	77 [69, 81]	0.11
Male	171 (57)	81 (54)	90 (60)	0.35
Body surface area, m^2^	1.59 [1.45, 1.75]	1.58 [1.43, 1.75]	1.61 [1.46, 1.75]	0.78
Body mass index	22.8 [20.2, 25.2]	22.7 [20.2, 25.6]	22.9 [20.4, 24.9]	0.92
NYHA classification				0.48
I	46 (15.3)	21 (14.0)	25 (16.7)	
II	213 (71.0)	109 (72.7)	104 (69.3)	
III	39 (13.0)	20 (13.3)	19 (12.7)	
IV	2 (0.7)	0 (0.0)	2 (1.3)	
Diabetes mellitus	130 (44)	53 (36)	77 (52)	0.01
Hypertension	168 (57)	75 (51)	93 (62)	0.07
Dyslipidemia	116 (39)	55 (37)	61 (41)	0.62
Ischemic heart disease	64 (21)	23 (15)	41 (27)	0.02
Atrial fibrillation	138 (46)	69 (46)	69 (46)	1.00
Pacemaker	10 (3)	4 (3)	6 (4)	0.75
Defibrillator	30 (10)	16 (11)	14 (9)	0.85
CRT	20 (7)	11 (7)	9 (6)	0.82
RASS inhibitor	194 (65)	100 (67)	94 (63)	0.55
ACE inhibitor	93 (31)	54 (36)	39 (26)	0.08
ARB	103 (34)	46 (31)	57 (38)	0.22
β blocker	258 (86)	131 (87)	127 (85)	0.62
MRA	132 (44)	74 (49)	58 (39)	0.08
SGLT2 inhibitor	54 (18)	23 (15)	31 (21)	0.29
Systolic blood pressure, mmHg	119 [104, 133]	120 [102, 133]	118 [104, 133]	0.68
Diastolic blood pressure, mmHg	67 [60, 75]	67 [59, 76]	67 [60, 73]	1.00
Heart rate,/min	71 [62, 80]	71 [60, 81]	71 [65, 79]	0.35
NT-proBNP, pg/mL	1220 [519, 2820]	1260 [513, 2835]	1165 [571, 2733]	0.93
eGFR, mL/min/1.73 m^2^	34 [28, 41]	37 [30, 42]	32 [26, 39]	<0.001
CKD classification				0.001
G1 to G3a	0 (0)	0 (0)	0 (0)	
G3b	200 (66.7)	114 (76.0)	86 (57.3)	
G4	90 (30.0)	35 (23.3)	55 (36.7)	
G5	10 (3.3)	1 (0.7)	9 (6.0)	
LVDd, mm	50 [45, 56]	50 [45, 56]	51 [45, 56]	0.38
LVDs, mm	36 [30, 45]	36 [29, 45]	36 [30, 45]	0.78
LVEF, %	45 [32, 58]	45 [32, 58]	46 [31, 58]	0.94
LVEF < 40%	121 (41)	64 (43)	57 (40)	0.61
Left ventricular mass index, gm/m^2^	117 [94, 140]	117 [92, 139]	117 [96, 143]	0.44
LA diameter, mm	44 [39, 49]	44 [38, 49]	44 [41, 49]	0.20
e’, cm/s	4.4 [3.5, 5.8]	4.5 [3.6, 5.9]	4.2 [3.4, 5.6]	0.21
E/e’	15.2 [11.6, 21.0]	15.2 [11.1, 18.8]	15.3 [11.9, 22.8]	0.27
Indoxyl sulfate, mg/mL	9.63 [5.85, 14.64]	5.85 [3.91, 7.70]	14.65 [11.40, 20.90]	<0.001

Categorical and continuous variables are described as n (%) and median [interquartile range], respectively. Abbreviations: ACE, angiotensin-converting enzyme; ARB, angiotensin receptor blocker; CRT, cardiac resynchronization therapy; LA, left atrial; LVDd, left ventricular diastolic diameter; LVDd, left ventricular systolic diameter; LVEF, left ventricular ejection fraction; MRA, mineralocorticoid receptor antagonists; NT-proBNP, N-terminal pro B-type natriuretic peptide; NYHA, New York Heart Association; RASS inhibitor, renin angiotensin aldosterone system inhibitor; SGLT2, sodium-glucose transport protein 2.

**Table 2:** The primary and secondary outcomes in each indoxyl sulfate group.

	Overall	Low Indoxyl Sulfate < 9.63 mg/mL	High Indoxyl Sulfate ≥ 9.63 mg/mL
n	300	150	150
Primary outcome	54 (18.0)	27 (18.0)	27 (18.0)
All-cause death	19 (6.3)	9 (6.0)	10 (6.7)
Cardiac death	13 (4.3)	4 (2.7)	9 (6.0)
Hospitalization for heart failure	43 (14.3)	22 (14.7)	21 (14.0)
Nonfatal myocardial infarction	0 (0)	0 (0)	0 (0)
Nonfatal stroke	2 (0.7)	0 (0)	2 (1.3)
Renal composite outcome	1 (0.3)	0 (0)	1 (0.7)

## Data Availability

Data are available upon request from the corresponding author.
